# Meniscus repair using mesenchymal stem cells – a comprehensive review

**DOI:** 10.1186/s13287-015-0077-2

**Published:** 2015-04-30

**Authors:** Hana Yu, Adetola B Adesida, Nadr M Jomha

**Affiliations:** Laboratory of Stem Cell Biology and Orthopaedic Tissue Engineering, University of Alberta, 3-021 Li Ka Shing Building, Edmonton, AB T6G 2E1 Canada; Division of Orthopaedic Surgery, Department of Surgery, 2D2.32 Department of Surgery, University of Alberta, Edmonton, AB T6G 2B7 Canada

## Abstract

The menisci are a pair of semilunar fibrocartilage structures that play an essential role in maintaining normal knee function. Injury to the menisci can disrupt joint stability and lead to debilitating results. Because natural meniscal healing is limited, an efficient method of repair is necessary. Tissue engineering (TE) combines the principles of life sciences and engineering to restore the unique architecture of the native meniscus. Mesenchymal stem cells (MSCs) have been investigated for their therapeutic potential both *in vitro* and *in vivo*. This comprehensive review examines the English literature identified through a database search using Medline, Embase, Engineering Village, and SPORTDiscus. The search results were classified based on MSC type, animal model, and method of MSC delivery/culture. A variety of MSC types, including bone marrow-derived, synovium-derived, adipose-derived, and meniscus-derived MSCs, has been examined. Research results were categorized into and discussed by the different animal models used; namely murine, leporine, porcine, caprine, bovine, ovine, canine, equine, and human models of meniscus defect/repair. Within each animal model, studies were categorized further according to MSC delivery/culture techniques. These techniques included direct application, fibrin glue/gel/clot, intra-articular injection, scaffold, tissue-engineered construct, meniscus tissue, pellets/aggregates, and hydrogel. The purpose of this review is to inform the reader about the current state and advances in meniscus TE using MSCs. Future directions of MSC-based meniscus TE are also suggested to help guide prospective research.

**Electronic supplementary material:**

The online version of this article (doi:10.1186/s13287-015-0077-2) contains supplementary material, which is available to authorized users.

## Introduction

The menisci are a pair of semilunar fibrocartilage structures well recognized to contribute to the maintenance of normal knee function, facilitating joint stability, load distribution, and joint lubrication. Importantly, partial or total meniscectomy predisposes the joint towards osteoarthritis (OA) [1-3]. Meniscus repair is of special interest to orthopedic clinicians and researchers because the menisci are frequently susceptible to injury in a diverse patient population [4-6].

Natural meniscal healing is limited. The outer third of the meniscus, or the red–red zone, is well vascularized and has a good healing capacity, while the intermediate red–white zone and the innermost white–white zone have poor intrinsic healing owing to their avascular nature [7,8]. Before it was known that the menisci play an essential role in cartilage protection and prevention of OA, partial or total meniscectomy was widely used to relieve the symptoms of meniscal injuries. However, since the dire consequences of such procedures had become widely recognized, more conservative approaches have been favored [9]. Nevertheless, the removal of damaged meniscus is inevitable in cases of serious meniscus injury. In such cases, attempts can be made to replace the damaged tissue with a functional substitute to restore function of normal meniscus. Although meniscus transplantation using allografts can achieve encouraging clinical results [10-14], concerns over many factors – tissue availability, mismatch of size and shape of graft and host, immunogenicity, disease transmission, and deterioration of the allograft’s mechanical properties after implantation – still persist [15-17]. To circumvent these problems, synthetic meniscus substitutes have been tested. Specifically, two acellular scaffolds – the collagen meniscus implant and Actifit® (Orteq Sports Medicine, London, UK) – are in clinical use. Both the collagen meniscus implant [18,19] and Actifit® [20] have been shown to improve pain and functional and radiological outcomes, but approximately one-third of collagen meniscus implant patients do not receive any benefit and improved, well-designed, long-term studies are needed to determine the efficacy and safety of collagen meniscus implantation [21]. Similarly, a current update on Actifit® notes that while preliminary published data appear promising, there are currently no medium-term or long-term data available for the scaffold [22].

An alternative approach to meniscus repair, especially for less severe lesions, focuses on promoting natural healing of the meniscus rather than replacing the damaged tissue. These techniques involve conduit treatment [23], abrasion therapy [24,25], and platelet-rich plasma (PRP) therapy [26]. As mentioned earlier, lesions of the inner regions of the meniscus do not heal spontaneously owing to their lack of vascularity. Conduit treatment supports healing of the avascular region by bridging the area of injury to the peripheral synovial tissues [23]. Similarly, abrasion therapy promotes vascular ingrowth to the avascular region by rasping the parameniscal synovium and the surface of the meniscus to attract synovial tissue and various cytokines to the injury site [24,25]. A less invasive method of treatment involves the use of PRP, an autologous, platelet-rich substance containing various growth factors that promote healing by enhancing meniscus cell proliferation, vascularization, and recruitment of fibroblasts and bone marrow-derived stem cells to the lesion [26]. Despite the benefits these techniques may offer, the therapies have significant drawbacks; a search for an innovative and effective treatment method therefore led to the advent of a new era – the era of meniscus tissue engineering (TE).

Cells constitute the backbone of TE. With respect to meniscus TE, meniscal fibrochondrocytes (MFCs), articular chondrocytes, and mesenchymal stem cells (MSCs) have all been tested successfully for their regenerative potential [27]. Among the various cell sources, MSCs are of special interest because of their multilineage plasticity towards a variety of mesenchymal tissues [28], potential immunomodulatory and anti-inflammatory properties [29,30], and extensive proliferative ability [29]. Furthermore, MSCs can migrate to the site of injury [31,32] and exert their reparative effects not only by replacing expired or damaged tissues, but also via trophic factors [33]. The purpose of this review is to outline the advances in meniscus repair using MSCs, and to present the current status and future directions of MSC-based meniscus TE. Methods are described in Additional file 1.

## Mesenchymal stem cell sources

MSCs can be isolated from various anatomical locations, with bone marrow-derived mesenchymal stem cells (BM-MSCs) used most commonly for meniscus TE, followed by synovium-derived mesenchymal stem cells (SMSCs). Other sources of MSCs included adipose-derived mesenchymal stem cells (ASCs) [34-36], meniscus-derived mesenchymal stem cells (MeMSCs) [34,37,38], and MSCs derived from other intra-articular and extra-articular tissues [34,39,40] such as muscles, ligaments, periosteum, and dermis (Table 1).

**Table 1:** **Classification based on mesenchymal stem cell type**

**Mesenchymal stem cell type**	**References**
Bone marrow	[34,39,41-81]
Synovium/synovial	[34,67,68,82-90]
Adipose	[34-36]
Meniscus	[34,37,38]
Others/not specified	[34,39,40,91]

The therapeutic potential of BM-MSCs has been well established by many *in vitro* and *in vivo* studies reporting that BM-MSCs promoted macroscopic and microscopic healing of meniscal defects [39,41-63]. Treatment of meniscal defects with BM-MSCs as described in these studies resulted in abundant extracellular matrix (ECM) production and, ultimately, in the production of meniscus-like fibrocartilage tissue that integrated well with the surrounding host tissue. In general, compared with their respective controls (no BM-MSCs), the newly generated tissue had improved mechanical properties [54-57]. Furthermore, BM-MSC groups showed chondroprotective effects. Alternatively, some studies are less supportive for the effects of BM-MSCs for meniscal healing [64-66], including less total meniscal healing in the lesions supplemented with exogenous fibrin clot and BM-MSCs [64]. Conversely, those menisci that did heal using BM-MSCs showed a more normal appearance with an increased and better oriented matrix. The authors concluded that cultured autologous BM-MSCs were not beneficial for enhanced meniscal healing despite a qualitative difference. Horse meniscal fragments inserted into a nude mouse model were used to investigate adhesion of two meniscal fragments with and without the addition of BM-MSCs to allogeneic fibrin [65]. Although subjective evaluations showed improved bonding and healing in BM-MSC-treated groups with increased vascular ingrowth as compared with their controls, all other parameters such as cell type, cell ingrowth, fibrous ingrowth, total bonding, and safranin O staining were not significantly different. The authors stated a concern of being underpowered with the use of eight horse menisci. Finally, the productivity and proliferation of human BM-MSCs were inferior to MFCs when cultured on nanofibrous scaffolds, although their biosynthetic outputs were similar when cultured in pellet form [66]. This result is contrary to the report made by the same group in an earlier study [48], which claimed that BM-MSCs can be used as an alternative to MFCs for meniscal TE. The absence of a preliminary differentiation period for the MSCs and use of a synthetic scaffold (poly-ε-caprolactone and poly(ethylene oxide)) could have been contributors to these poor MSC results.

The therapeutic capacity of SMSCs has been demonstrated, although studied to a less extensive degree than that of BM-MSCs [67,82-88]. Compared with their respective controls, the quantity and quality of regenerated tissue was significantly greater in those groups treated with SMSCs. Importantly, it has been shown that MSC numbers in synovial fluid increased after a meniscus injury in human subjects, suggesting an important role for SMSCs in meniscus healing [86,87]. Injected SMSCs have been shown to adhere to created meniscal defects and differentiate into chondrocytes *in vivo* after injection into a rat joint model. Unfortunately, sufficiently sized defects were not created because healing was noted in the control group that did not have cells injected. The lack of difference between the SMSC-treated group and the control group using histological analyses is therefore not informative [89]. Meniscus regeneration by syngeneic, minor mismatched, and major mismatched transplantation of SMSCs has been investigated in a rat model, and it was observed that syngeneic and minor mismatched transplantation of SMSCs resulted in improved meniscus healing compared with major mismatched transplantation [90]. The degree of immunocompatibility between host and donor cells is thus an important factor that can have a profound effect on the regenerative potential of stem cells. As mentioned earlier, one potential benefit of MSCs is their immunosuppressive property. However, this study advises that when using allogeneic SMSCs, the MSC histocompatibility antigens should be closely matched to the recipients’ antigens to achieve best results. This could be a potential difference between BM-MSCs and SMSCs.

Other types of MSCs, such as ASCs [35,36] and MeMSCs [37,38], have been used successfully to promote the regeneration of meniscus *in vivo*. However, there is a need to further characterize the stemness of MeMSCs, since their isolation protocol is not different from the widely-used native MFC isolation method, and native MFCs have been shown to display characteristics similar to MSCs [92]. In addition, dermis-isolated adult stem cells produce meniscus-like tissue with robust mechanical properties [40]. The effectiveness of periosteum-derived MSCs has been tested [39], but the periosteal graft containing osteochondroprogenitor cells was deemed ineffectual as a meniscal substitute.

The healing potential and gene expression profiles of MSCs from different tissues have been investigated [34,67,68]. No notable morphological differences of regenerated meniscus between rat BM-MSC and SMSC groups were reported, with the SMSC group performing better than the BM-MSC group in terms of the type II collagen COL2 expression and electron microscopic features [68]. Moreover, hierarchical clustering analysis revealed that gene expression of rat meniscal cells was closer to that of SMSCs relative to BM-MSCs. The same researchers confirmed superior *in vitro* proliferation of rabbit SMSCs compared with BM-MSCs [67]. Gene expression profiles of human MSCs derived from intra-articular and extra-articular tissues also found that MSCs from intra-articular tissues (such as synovium, meniscus, and ligament) and chondrocytes were closer to each other than those derived from extra-articular tissues (such as muscle, extra-articular adipose tissue, and bone marrow) [34]. There is thus the potential for certain MSCs to be more effective in meniscal healing.

In summary, many different sources of MSCs have been tested and shown to be effective with respect to their therapeutic potential, but there exists a clear imbalance in research efforts between different types of MSCs. Gene expression analyses showed that intra-articular tissue-derived MSCs such as SMSCs are genetically closer to MFCs and MeMSCs, indicating that a more in-depth investigation of intra-articular tissue-derived MSCs will be beneficial. Currently, no definite answer regarding the most optimal source of MSCs for meniscus repair exists and further exploration of different MSC sources and research on their effectiveness is encouraged.

## Different animal models

### Small animal models

The murine model has been investigated by different research groups. The *in vitro* chondrogenic potential of rat BM-MSCs when cultured in decellularized scaffolds from a normal rat meniscus has been demonstrated [46]. BM-MSC seeded scaffolds showed increased expression of ECM after 4 weeks and stiffness increased with time, resulting in a neotissue approximating a normal meniscus. The same researchers used the decellularized meniscus scaffold model and demonstrated the efficacy of the scaffold when repopulated with rat BM-MSCs for meniscal transplantation [51]. In addition, BM-MSCs have been shown to proliferate in the avascular organ culture model and contribute to meniscal healing by producing an abundant ECM, when full-thickness circular defects of rat meniscal explants were filled with BM-MSCs [44]. *In vivo* experiments using various modalities of MSC delivery – intra-articular injection [38,47,60], scaffolds [51], and aggregates [84] – have also successfully demonstrated the therapeutic potential of MSCs. When a large number of BM-MSCs (that is, 1.0 × 10^7^ cells) were injected into multiple-tissue-injured rat knee joints, transplanted cells effectively mobilized to the injured tissue and *in situ* MSC differentiation was observed [47]. Likewise, rat or human BM-MSCs injected into rat knee joints, after a hemi-meniscectomy had been performed resulted in promotion of rat COL2 synthesis in the regenerating meniscus with chondroprotective effects [60]. Similar results were seen when human MeMSCs were injected into rat joints whose anterior half of the medial meniscus had been removed [38]. Even though MSCs have been shown to home to injury sites, the effectiveness of intra-articular injection technique should be investigated further [47]. Transplantation of aggregates of SMSCs regenerated meniscus more effectively than intra-articular injection of SMSCs [84], possibly by increasing chondrogenic potentials of SMSCs, by applying SMSCs more directly to the meniscal defect, and by maintaining cell viability longer compared with suspended SMSCs.

In the leporine model, MSCs administered directly into meniscal defects or intra-articularly injected into damaged joints resulted in promotion of fibrocartilaginous tissue regeneration that was grossly and histologically meniscus like [35,37,42,67,68,83] and effectively inhibited progression of articular cartilage degeneration [37,68,83]. Various types of scaffolds, namely the type I collagen COL1 [39] and composite scaffolds [41,49,61], have been used successfully in combination with MSCs to treat meniscal defects: scaffolds containing MSCs produced more abundant regenerative tissue that was superior in microscopic and macroscopic quality. Caution is needed when extrapolating the results to humans. Interspecies differences have to be acknowledged [37,38,41,49,60,61,67,68,83], especially considering the facts that animal menisci are smaller, have different load cycles and gait pattern, and have spontaneous healing potential (Table 2).

**Table 2:** **Classification based on animal model used**

**Animal model**	**References**
Human	[34,36,38,50,54,56,60,63,66,70-73,76-79,86,87]
Equine	[62,65]
Canine	[45]
Ovine	[54,58,59]
Bovine	[48,53-55,57,69,74,75,80,91]
Caprine	[40,43,64,81]
Porcine	[52,82,85,88]
Leporine	[35,37,39,41,42,49,61,67,68,83]
Murine	[38,44,46,47,51,60,65,84,89,90]

### Large animal models

MSCs cultured *in vitro* via chondrogenic passaging, self-assembly processing and micromass formation [40], as well as in hydrogel [69] and in various types of scaffolds [48,54,55,57], have been shown to facilitate meniscal repair in large animals. MSCs directly applied to the injury site [52,58], injected intra-articularly [43,59,88], or transplanted as scaffold-free tissue-engineered construct [82,85] contributed to the meniscal structure and function restoration. Application of MSCs resulted in secure defect filling with fibrocartilaginous neotissue, showing good integration. The repaired tissue showed a significant biomechanical improvement compared with controls; however, mechanical properties were still inferior to normal tissue [52,82]. Nevertheless, MSCs prevented degeneration of meniscus and retarded the progression of OA.

An *in vivo* canine model demonstrated that injection of autologous BM-MSCs into the red–white zone tear of lateral menisci markedly improved meniscal wound healing compared with non-injected, control menisci [45]. Subcutaneously implanted equine meniscal sections with fibrin glue (MSC-free or with MSCs) into nude mice showed no statistically significant objective results but subjectively enhanced meniscus repair was seen in constructs treated with MSCs/fibrin compared with constructs treated with fibrin alone [65]. Subsequently, the same researchers performed a prospective case series using an *in vivo* equine model [62]. A total of 15 to 20 million autologous BM-MSCs were injected into the affected joints and were followed for 2 years after the treatment. Outcomes were evaluated using lameness evaluation and survey. Overall, 42% of the horses returned to or exceeded their previous work level, 33% returned to work, although not at previous standards, and 8% failed to return to work. These results were compared with previous reports that treated meniscal defects with arthroscopy alone [93,94], and intra-articular administration of MSCs was overall a safe procedure that can bring about better clinical outcomes than arthroscopic surgery alone for meniscal lesions (Table 2).

### Human models

For *in vitro* experiments, human MSCs have been cultured mainly using various types of scaffolds and pellets (Table 2). Overall, culturing on scaffolds led to the formation of meniscus-like tissue that showed significantly higher tensile strength, and axial/radial compressive moduli comparable with those of native meniscus [54,56]. Pellet cultures have been employed primarily for co-culturing of MSCs with other cell types [70-73], to be discussed later in this review.

The role of MSCs in meniscus repair has been examined in humans *in vivo*. As discussed earlier, the number of MSCs in synovial fluid increased after meniscus injury while the total MSC colony number per synovial fluid volume was positively correlated with the postinjury period [86,87], suggesting that MSCs play an important role in meniscus healing. Clinically, autologous MSCs have been intra-articularly injected into a patient suffering from meniscus injury – as documented in a case study where 22.4 million precultured autologous MSCs along with 1 ml nucleated cells (isolated from fresh bone) and 1 ml 10% v/v platelet lysate were intra-articularly injected into a 46-year-old male patient suffering from degenerative knee changes [50]. The patient received two additional platelet lysate injections (supplemented with 1 ml 10 ng/ml dexamethasone) at weeks 1 and 2 post-transplantation. Using radiographic imaging, pre-treatment and post-treatment subjective visual analog pain scores, and physical therapy assessments, it was concluded that the percutaneous injection of MSCs into the ‘knee with symptomatic and radiographic degenerative joint disease resulted in significant cartilage and meniscus growth, decreased pain, and increased joint mobility’ [50]. However, because the patient received the platelet lysate with low-dose steroid twice post-procedure, it is difficult to conclude that the MSC application was the sole contributor to the physiological and functional improvement. Furthermore, these results were only 3 months post-injection.

Similarly, Pak and colleagues reported a case study in which a percutaneous injection of autologous ASCs along with PRP, hyaluronic acid, and CaCl_2_ (collectively termed an ASC mixture) promoted the repair of a grade II meniscal tear in a 32-year-old female [36]. The ASC mixture was injected into the medial tibiofemoral joint and into the medial inferior retropatellar joint on the day of ASC collection. On the third and seventh days after the initial injection, the patient received another dose of PRP with CaCl_2_ and hyaluronic acid. On the 14th day after the initial injection, a mixture of PRP, CaCl_2_, and a low-dose dexamethasone was injected. Finally, the last dose of PRP with CaCl_2_ was given on day 28. A comparison of the pain scores (using a functional rating index and visual analog scale) before and 3 months after the treatment showed significant symptom improvements, and a near-complete repair of the torn meniscus was seen on repeated magnetic resonance imaging. Because the past treatment attempts with PRP and hyaluronic acid injections had all failed, the authors reasoned that the addition of ASCs led to the clinical improvements.

A randomized, double-blind, controlled study included 55 patients who underwent a partial medial meniscectomy and were randomly assigned to three different treatments: Group A, injection of 50 million allogeneic MSCs suspended in sodium hyaluronate/human serum albumin/PlasmaLyte A(Baxter Healthcare Corporation) (*n* = 18); Group B, injection of 150 million allogeneic MSCs suspended in the same hyaluronate solution (*n* = 18); or Group C, injection of sodium hyaluronate solution control (*n* = 19) [63]. Patient assessments were performed up to 2 years postoperatively and included safety evaluation, meniscus regeneration, overall knee joint condition, and clinical outcomes. Sequential magnetic resonance imaging assessed the meniscus volume, cartilage degeneration, and ectopic tissue formation. Knee pain and function were evaluated using a visual analog scale and the self-assessment Lysholm knee scale. No adverse events leading to study termination were observed. Respectively, 24% and 6% of the patients in Groups A and B experienced a significant meniscal volume gain (defined *a priori* as a 15% threshold) as defined on magnetic resonance imaging at 1 year post-implantation, although this decreased to 18% and 0% respectively by 2 years post-implantation. No patient from control Group C met the 15% threshold for increased meniscal volume. Furthermore, a greater proportion of those with OA changes experienced a reduction in pain following the treatment with MSCs relative to the control group. This study demonstrated that high doses of allogeneic MSCs can be safely injected into the knee-joint without ectopic tissue formation and that treatment with MSCs may lead to *de novo* meniscus tissue regeneration.

## Current strategies and future directions

### Undifferentiated versus differentiated MSCs

The choice between undifferentiated and differentiated MSCs for meniscus repair can influence the outcome of MSC therapy (Table 3). Insertion of a human BM-MSC-seeded collagen scaffold between two ovine fibrocartilage discs and subsequent histological analyses revealed that MSCs which had been chondrogenically differentiated using transforming growth factor beta 1 (TGF-β1) led to significantly less integration while the undifferentiated MSCs showed significant integration with the ovine meniscal surface [54]. Conversely, when MSCs cultured in chondrogenic media or in basal media were intra-articularly injected into an ovine model (in which OA was induced via total medial meniscectomy and resection of the anterior cruciate ligament), the chondrogenic media group demonstrated a significant regeneration of fibrocartilage tissue while the basal media group showed only evidence of scarring [59]. Finally, treatment of 4 mm longitudinal meniscal tears in the avascular zone of leporine lateral menisci with undifferentiated BM-MSCs resulted in defect filling with repair tissue, but also partial disintegration [61]. Meniscal lesions treated with differentiated MSCs led to a near-complete filling of the defects with dense repair tissue. The researchers suggested that the necessity of treatment with undifferentiated or differentiated MSCs seemed dependent on the nature of meniscal defects.

**Table 3:** **Classification based on method of mesenchymal stem cell delivery/culture**

**Culture system**	**References**
Direct application	[35,42,45,52,58,64,67,81,84]
Fibrin glue/gel/clot	[42,44,52,53,64,65]
Intra-articular injection	[36-38,43,47,50,59,60,62,63,68,83,88-90]
Scaffold	[39,41,48,49,54-57,61,66,74-78,80,91]
Tissue-engineered construct	[82,85]
Meniscus tissue	[46,51]
Pellets or aggregates	[40,70-73,84]
Hydrogel	[69]
Others	[34,40,79,86,87]

### Mechanical stimulation and perfusion

Menisci are constantly exposed to a variety of mechanical stresses. Several studies have attempted to mimic the mechanical stresses using dynamic bioreactors [74-77]. Baker and colleagues [74] and Nerurkar and colleagues [75] observed that application of dynamic mechanical stimulation to MSC-seeded scaffolds increased the total collagen content and promoted the maturation of MSC-laden constructs, although they noted that dynamic loading can lead to marked loss in proteoglycan content. Baker and colleagues documented a significant improvement in mechanical properties of mechanically stimulated constructs, while Nerurkar and colleagues showed that dynamic culture failed to produce any significant gain in mechanical function relative to controls. Both groups agreed that additional work was necessary to interpret the mechanical implications of dynamic bioreactors, especially to reduce the gap between the mechanical property of MSC-loaded samples and native meniscus.

More recent studies have investigated the effect of applying a variety of mechanical stimulation on the structural and functional properties of TE constructs. Petri and colleagues [77] and Liu and colleagues [76] cultivated human BM-MSCs on scaffolds under a static condition, with continuous perfusion (10 ml/minute), or with both continuous perfusion (10 ml/minute) and mechanical stimulation (10% cyclic compression at 0.5 Hz). Overall, samples under perfusion or with both perfusion and mechanical stimulation showed enhanced ECM accumulation and mechanical properties compared with the static culture group. However, unlike Petri and colleagues who found 8 hours of daily mechanical stimulation with continuous perfusion beneficial, Liu and colleagues found that 8 hours of continuous stimulation had a negative effect. Instead, Liu and colleagues reported continuous perfusion coupled with intermittent mechanical stimulation (that is, four times daily for 2 hours each time with 4 hours of rest in between) to be more beneficial. This discrepancy mandates further investigation.

The combined results from these studies suggest that perfusion and mechanical loading can enhance the quality of TE meniscus. While the mechanical properties of TE constructs cultivated under various perfusion/mechanical loading conditions improved compared with constructs under static conditions, functional properties were inferior to those of natural meniscus. Further optimization of a perfusion/mechanical stimulation protocol is therefore required to produce MSC-seeded implants suitable for clinical application.

### A three-dimensional niche: hydrogel and aligned nanofibrous scaffolds

*In vivo*, the ECM provides scaffolding for cells. To mimic this three-dimensional culture environment, various biomaterials have been used to create scaffolds. Hydrogels containing different proportions of chondroitin sulfate and bone marrow have been synthesized and examined [69]. MFCs were suspended in the hydrogels that were cultured in basal meniscus medium until harvest. Chondroitin sulfate–poly(ethyleneglycol) was used as a control. Chondroitin sulfate–bone marrow hydrogels supported the survival, proliferation, and metabolic activity of MFCs, unlike the chondroitin sulfate–poly(ethyleneglycol) control. High proportions of bone marrow stimulated proliferation and migration of cells while increasing the chondroitin sulfate content stimulated ECM production and increased adhesive strength, demonstrating that MSCs can be used in conjunction with other materials and cells to enhance cell function and matrix production for meniscus healing.

Other advancements in scaffolding technology include the development of polyurethane scaffolds coated with a thin layer of a novel cross-linked gelatin hydrogel system [78], and aligned nanofibrous scaffolds [48,57,66,91]. Modified polyurethane films were developed to provide specific cell binding sites for MSCs, allowing for adequate MSC adhesion and spreading across scaffolds [78]. Aligned nanofibrous scaffolds were engineered to help promote maturation of MSCs and accumulation of ECM in an organized fashion that improves mechanical function of tissue-engineered constructs [48,57]. The orientation and shape of cells, DNA content, total collagen content, and mechanical properties of MSC-laden scaffolds were shown to be dictated by scaffold architecture: aligned nanofibrous scaffolds provided a three-dimensional micro-pattern for guiding short-term and long-term organization of MSCs and newly deposited ECM, which contributed to the functional maturation of the engineered meniscal constructs [48]. Electrospun scaffolds of different fiber sizes – small (70 to 486 nm) versus large (221 to 1,461 nm) – have been compared, intending to explore how topographic cues affect maturation of MSCs [91]. MSCs cultured in small fiber scaffolds showed a rounded/polygonal morphology, while those cultured in large fiber scaffolds elongated in the fiber direction. Many factors are involved in optimizing meniscus regeneration, including the scaffold shape, scaffold material, material orientation, and cell content.

### Co-culture and hypoxic culture

MSCs secrete a wide range of trophic factors that can interact with nearby cells to promote healing [33,95,96]. Although the mechanism of interaction has not been explored, direct co-cultures of MSCs and MFCs formed a neotissue with enhanced production of the functional ECM of meniscus and reduced hypertrophy of MSCs relative to pure MSCs or MFCs [70-73]. Additionally, hypertrophic differentiation of MSCs was better suppressed when MSCs were co-cultured with MFCs from the outer meniscus relative to inner MFCs [71]. Furthermore, co-cultures of MFCs and MSCs in different proportions have been examined. Comparison of bilaminar cell pellets created using MFCs and MSCs at varying ratios of 1:0, 3:1, 1:1, 1:3, and 0:1 determined that co-cultures of MFCs with MSCs in a 3:1 ratio yielded the highest levels of COL1 and glycosaminoglycan production, as well as the lowest levels of hypertrophic genes [70]. Saliken and colleagues [71] and Chowdhury and colleagues [72] produced promising results using cell pellets consisting of MFCs and BM-MSCs in a 1:3 ratio, a ratio shown to reproducibly result in enhanced matrix formation [73]. This discrepancy over the exact ratio of a co-culture (that is, MFC:MSC ratio of 3:1 in Cui and colleagues [70] and of 1:3 in Matthies and colleagues [73]) mandates further investigation; however, the fact that one of the benefits of a co-culture derives from reducing the need for MFC expansion must be considered in future research.

Dermis isolated adult stem cells have been used to show that hypoxia may be an important factor for enhancing cartilage-like properties of MSC-engineered constructs [40,73]. The use of a co-culture system instead of a monoculture system and hypoxic culture conditions may help improve the quality of TE meniscus and may mitigate the need to expand MFCs.

### Genetic profiling and modification of MSCs

MSC gene expression has been investigated in an attempt to better understand MSC-driven meniscus TE. cDNA microarrays were analyzed to compare 92,160 gene expression patterns in articular and fibrocartilage tissues with 669,160 measurements of genes expressed in 29 other human tissues [79]. Results showed that both hyaline and fibrocartilage tissues share very similar gene expression patterns, and that among several mesodermal tissues only bone marrow and nerve tissues yielded expression patterns comparable with those of cartilage. Eleven genes specific to both types of cartilage were identified. These 22 genes can potentially serve as cell markers during chondrogenesis of multipotent MSCs. It would be interesting to examine whether the expression of these markers can be induced in MSCs to destine pluripotent cells to a specific differentiation pathway. This approach to meniscus engineering could be especially useful because the meniscus exhibits a combination of articular and fibrocartilaginous phenotypes [97].

Genetic modification of MSCs has been tested for its efficacy. MSCs have been engineered to directly deliver TGF-β1 [80] and human insulin-like growth factor-1 to the site of meniscal defect [81]. Transplantation of collagen–glycosaminoglycan copolymer scaffolds seeded with TGF-β1-transduced MSCs into 5 mm longitudinal tears in the avascular zone of the meniscus resulted in effective lesion filling with repair tissue after 3 weeks of *in vitro* culture [80]. Stimulation with MSC-secreted TGF-β1 also increased the cellularity, deposition of proteoglycans and COL2, and enhanced expression of meniscal genes. The authors recognized that an uncontrolled ubiquitous expression of TGF-β1 can lead to severe joint fibrosis and detrimental systemic effects. Future studies should therefore investigate methods of localized delivery of TGF-β1 within meniscal defects. Similarly, positive aspects of human insulin-like growth factor-1-transfected BM-MSCs in repairing a full-thickness model of meniscal defect by delivering biologically effective concentrations of human insulin-like growth factor-1 have been demonstrated [81]. Long-term studies are required to monitor the safety and fate of these genetically modified MSCs. Furthermore, more research is needed to explore and determine the type and amount of growth factors (or combination of factors) most appropriate for meniscus engineering.

## Conclusion

Meniscus injury is common and can lead to degenerative joint changes, given the current state of medical care, unless significant advancements are made in meniscus repair and regeneration technologies. TE aims to restore the structural and functional characteristics of meniscus by reconstructing the unique meniscus architecture. MSCs are a useful cell source for meniscus TE. The role and effectiveness of MSCs isolated from different anatomical locations and animal models have been investigated using a wide range of culture/delivery techniques. A comprehensive review of the literature suggests that MSCs possess an intrinsic therapeutic potential that can directly and indirectly contribute to meniscus healing. It is interesting that despite the positive and promising results of MSC use in meniscus repair, few techniques have reached clinical application. The reason for this is unclear; however, it may reflect the complexity of the tissue itself, partial vascularity, interspecies variability of meniscus, multiple cell types within the tissue, and so forth. Unfortunately, current research has only superficially examined most of these avenues and extensive work is required to identify the best MSC source and optimize the application of these cells. The future of meniscus TE lies in developing ways to maximally exploit the healing capacity of MSCs. Only with further advancements in meniscus-driven TE can MSCs be safely and effectively applied in clinical settings to help repair meniscus defects and to prevent or slow the progression of OA.

## Additional file

Additional file 1:
**Methods.**

## Abbreviations

ASC: Adipose-derived mesenchymal stem cell
BM-MSC: Bone marrow-derived mesenchymal stem cell
ECM: Extracellular matrix
MeMSC: Meniscus-derived mesenchymal stem cell
MFC: Meniscal fibrochondrocyte
MSC: Mesenchymal stem cell
OA: Osteoarthritis
PRP: Platelet-rich plasma
SMSC: Synovium-derived mesenchymal stem cell
TE: Tissue engineering
TGF-β1: Transforming growth factor beta 1
