# Supplementary material for: Meniscus repair using mesenchymal stem cells – a comprehensive review
Source: Stem Cell Res Ther. 2015 Apr 30;6(1):86. doi: 10.1186/s13287-015-0077-2 (PMC4415251; doi:10.1186/s13287-015-0077-2)
Supplement: Additional file 1: — Methods. [file 13287_2015_77_MOESM1_ESM.docx]

Additional file 1

Methods{Level 1 heading}

A comprehensive search (see following sections for search strategies) of the English literature was performed using Medline, Embase, Engineering Village (Compendex), and SPORTDiscus. English articles were identified using various combinations of the following keywords: mesenchymal, bone marrow cell, stromal cell, progenitor cell, stem cell, meniscus, and knee meniscus. Specific search steps performed within each database are outlined within this additional file. A total 325 articles, of which 95 were exact duplicates, were identified through database searching. After the deletion of duplicates, 286 results remained. These articles included a wide range of scientific reports including original research papers, review articles, conference proceedings and abstracts, and editor’s notes. Close duplicates were screened further for additional elimination. After subjective assessment for inclusion, 95 results were identified as relevant to the topic of our interest. Of these, 55 original research articles were closely reviewed, and they were categorized based on the type of mesenchymal stem cell used (Table 1), the animal model used (Table 2), and the method of mesenchymal stem cell delivery/culture (Table 3). Within each category, an article may have been included in more than one subset. In addition to the studies identified using the search strategies outlined here, two additional recently published studies (Hatsushika and colleagues; Pak and colleagues) were also included.

MEDLINE search{Level 2 heading}

Database: Ovid MEDLINE^®^ In-Process & Other Non-Indexed Citations, Ovid MEDLINE^®^ Daily and Ovid MEDLINE^®^ <1946 to present>

Search strategy:

1. exp Mesenchymal Stromal Cells/ or mesenchymal stem cell*.mp.
2. exp Mesenchymal Stromal Cells/ or mesenchymal stem cell*.mp. or exp Bone Marrow Cells/
3. (bone marrow adj2 stromal).mp.
4. (meniscus or menisc*).mp. [mp=title, abstract, original title, name of substance word, subject heading word, keyword heading word, protocol supplementary concept word, rare disease supplementary concept word, unique identifier]
5. 1 or 2 or 3
6. 4 and 5
7. limit 6 to English language

Output: 88 results

Embase search{Level 2 heading}

Database: Embase <1974 to 2014 week 21>

Search strategy:

1. exp Mesenchymal Stromal Cells/
2. (mesenchymal adj2 (cell* or stromal or progenitor* or stem)).mp.
3. (bone marrow adj2 stromal).mp. [mp=title, abstract, subject headings, heading word, drug trade name, original title, device manufacturer, drug manufacturer, device trade name, keyword]
4. 1 or 2 or 3
5. (meniscus or menisc*).mp. [mp=title, abstract, subject headings, heading word, drug trade name, original title, device manufacturer, drug manufacturer, device trade name, keyword]
6. 4 and 5
7. exp knee meniscus/
8. exp bone marrow cell/ or exp mesenchymal stem cell/
9. 4 or 8
10. 5 or 7
11. 9 and 10
12. limit 11 to English language

Output: 187 results

Engineering Village (Compendex) search{Level 2 heading}

Database: Engineering Village (Compendex) <1896 to 2015>

Search strategy:

(((((menisc*) WN ALL) AND ((mesenchy* or "bone marrow") WN ALL))) AND ({english} WN LA))

Output: 41 results

SportDiscus search{Level 2 heading}

Database: SportDiscus with Fulltext <1995 to 2014>

Search strategy:

Mesenchymal AND menisc*

Output: nine results
